# Response to ‘On the importance of understanding physiology when estimating energetics in cetaceans’

**DOI:** 10.1242/bio.023143

**Published:** 2017-02-08

**Authors:** A. Fahlman, J. van der Hoop, M. J. Moore, G. Levine, J. Rocho-Levine, M. Brodsky

**Affiliations:** 1 Fundación Oceanogràfic, c/Gran Vía Marqués del Turia 19, 46005, Valencia, Spain; 2 Massachusetts Institute of Technology – Woods Hole Oceanographic Institution Joint Program in Oceanography, 77 Massachusetts Ave, Cambridge, MA 02139, USA; 3 Biology Department, Woods Hole Oceanographic Institution, 266 Woods Hole Rd, Woods Hole, MA 02543, USA; 4 Dolphin Quest, Oahu, 5000 Kahala Ave, Honolulu, HI 96816, USA; 5 V.M.D. Consulting, Miami, FL 33138, USA; 6 Texas A&M University – Corpus Christi, 6300 Ocean Drive, Corpus Christi, TX 78412, USA

## Abstract

**Summary:** Our paper highlights how temporal changes in tidal volume and the oxygen exchange ratio significantly affect the accuracy of models that use only breathing frequency to estimate metabolic rate.

We are grateful for the interest in our paper by two eminent physiologists and hope this response to their comments will clarify the objectives of our paper. The analysis in Fahlman et al. (2016) was not intended to provide an accurate method to estimate field metabolic rate (FMR) in large mysticetes; the objective was to measure the dynamic changes in physiology associated with recovery from exercise and show that they are important to consider when estimating FMR. While static averages can provide useful estimates of FMR for a variety of situations, these need to be appropriately selected. For example, we illustrate that it is not possible to use selected average values chosen from excised tissues or resting animals (as in Blix and Folkow, 1995) to provide meaningful estimates of FMR for animals at different activities (i.e. the dolphins in our study). Our study highlights the importance of temporal variation in physiological models: the Blix and Folkow (1995) estimates rely on the assumption that only breathing frequency (*f*_R_) changes with activity, while we argue that both the tidal volume (VT) and mixed lung O_2_ content also vary with activity and recovery from a dive (Ridgway et al., 1969). Including this variation in all three parameters reduces temporal uncertainty in the same conceptual model (see Eqn. 1 in Fahlman et al., 2016).

Mathematical models are important tools in eco-physiology as they can create a framework with which to investigate complex physiological problems. It is therefore important to evaluate and revise these models as new information is gained or technological advancements are made. A recent example is how a theoretical model developed to improve our understanding of how mammalian gas exchange is altered during breath-hold diving (Fahlman et al., 2006) was repeatedly updated as new information became available (Fahlman et al., 2009; Hodanbosi et al., 2016). In this study, improved parameter estimates illustrated discrepancies in previous models and allowed us to identify the sensitivity of the system to specific inputs. Similarly, it is not surprising that our Model C (Fahlman et al., 2016) provided the best estimates in our study species, as empirical data from those dolphins were used to revise the input parameters. By doing so, we illustrated how the use of our data in Model C accounts for empirical uncertainties and temporal variation, as we were able to measure aspects of the system (VT and O_2_ content) that are difficult to record in a free-swimming cetacean, thus highlighting the dynamic nature of recovery from exercise.

Estimating FMR in large whales is not a straightforward exercise as there is limited information available for use with Eqn 1. The main critique in Folkow and Blix's Correspondence is that we inappropriately apply allometric relationships within the order *Cetacea*. While we agree that species-specific parameters and variables should be used when available, many theoretical models use estimates from closely related species. For example, even Blix and Folkow themselves (Blix and Folkow, 1995) used mixed pulmonary O_2_ from the harbor porpoise and bottlenose dolphin for their estimated O_2_ exchange ratio in minke whales, and picked a value for VT to be 60% of total lung capacity (TLC) while the measured VT was from 25-60% of TLC, which resulted in a metabolic rate that was 2×Kleiber (Wahrenbrock et al., 1974). Dolphin (1987) used a value for VT that was 80% of the vital capacity (VC) estimated from the bottlenose dolphin and pilot whale, resulting in surface and diving metabolic rates for humpback whales that were 6–10×Kleiber. Finally, Armstrong and Siegfried used an allometric equation for terrestrial mammals to estimate VC and multiplied this by 80% to estimate VT in minke whales, which resulted in daily metabolic rates around 3–4×Kleiber (Armstrong and Siegfried, 1991). In our study, we showed that both VT and Δ*O_2_* are probably significantly lower compared to the estimates used in these previous studies and that these parameters vary with both activity level and recovery time.

In their Correspondence, Folkow and Blix further argue that our Model C results in a resting metabolic rate for a 4000 kg minke whale (∼7.3 m) that is approximately 0.74×Kleiber. Their argument assumes that the physiology of all mysticetes is comparable, so we will follow this assumption for the following counter-argument. We would like to point out a few necessary corrections to this estimate. Our data for resting VT is 37% of estimated TLC (TLC_est_; Fahlman et al., 2011; Kooyman, 1973) or 103 l. With an O_2_ exchange ratio of 4.92% and respiration rate of 1.38 breaths min^−1^ (estimated from figures 4 and 5 in Wahrenbrock et al., 1974) the metabolic rate would be 7.0 l O_2_ min^−1^. A similar calculation using the assumptions by Blix and Folkow (1995) results in a value approximately 3.4×Kleiber or 68% higher than the measured value in Wahrenbrock (1974) for a gray whale. For a sleeping, swimming, and feeding minke whale (*f*_R_=0.59–0.89, *M*_b_=4000 kg), the estimate by Blix and Folkow (1995) would be approximately 2.0 to 2.9×Kleiber. Using our results where we measured the changes in continuous O_2_ uptake during recovery from exercise, and assuming that the first breath represents a close estimate of the instantaneous O_2_ consumption rate during steady-state exercise performed by the dolphins, we would get a VT of 146 l (53% of TLC_est_=278 l) and ΔO_2_ (6.6%). Thus, the O_2_ consumed per breath would be approximately 9.6 l, or 1.4×Kleiber (7 l O_2_ min^−1^) for a cruising minke whale.

The point of this exercise is to show how new data can be used to improve models by allowing us to evaluate past efforts and to illustrate their limitations. We agree with Folkow and Blix that we must use ‘proper physiological insight’ when considering parameter assumptions and application across species groups. The basic models A and B (Armstrong and Siegfried, 1991; Blix and Folkow, 1995; Dolphin, 1987) are widely applied across marine mammal species (e.g. Christiansen et al., 2014; Williams and Noren, 2009) and we encourage their evaluation (e.g. Fahlman et al., 2016; Roos, 2015) as they are applied to new species and in light of new developments in physiological research. We therefore argue that it is not possible to use static average values to estimate FMR for a range of activity levels to estimate metabolic rate from *f*_R_ with two unknown variables that are known to vary with exercise. It is well known that VT, *f*_R_, and ΔO_2_ vary with exercise and following diving (Fahlman et al., 2008, 2016; Kooyman et al., 1973; Ponganis et al., 1991, 1990; Reed et al., 1994, 2000; Ridgway et al., 1969), and we argue that this has to be accounted for to improve estimates of FMR.

We believe that our study (Fahlman et al., 2016) has helped provide insight into the dynamic nature of cardiorespiratory physiology of cetaceans, and that future studies will help improve our understanding. We agree with Folkow and Blix that our study has limitations; however, this discussion clearly shows how and why we need to be willing to evolve our understanding of physiology.
